# Fibrinogen Structural Changes and Their Potential Role in Endometriosis-Related Thrombosis

**DOI:** 10.3390/antiox13121456

**Published:** 2024-11-27

**Authors:** Eleonora Fini, Flavia Rita Argento, Serena Borghi, Elvira Giurranna, Francesca Nencini, Michela Cirillo, Cinzia Fatini, Niccolò Taddei, Maria Elisabetta Coccia, Claudia Fiorillo, Matteo Becatti

**Affiliations:** 1 Department of Experimental and Clinical Biomedical Sciences “Mario Serio”, University of Firenze, 50134 Firenze, Italy; eleonora.fini@unifi.it (E.F.); flaviarita.argento@unifi.it (F.R.A.); serena.borghi@unifi.it (S.B.); elvira.giurranna@unifi.it (E.G.); francesca.nencini@unifi.it (F.N.); michela.cirillo@unifi.it (M.C.); niccolo.taddei@unifi.it (N.T.); mariaelisabetta.coccia@unifi.it (M.E.C.); claudia.fiorillo@unifi.it (C.F.); 2 Centre for Assisted Reproductive Technology, Division of Obstetrics and Gynaecology, Careggi University Hospital, 50134 Florence, Italy; cinzia.fatini@unifi.it

**Keywords:** endometriosis, thrombosis, fibrinogen, oxidative stress

## Abstract

Endometriosis (EM), a chronic inflammatory condition predominantly affecting women of reproductive age, has been linked to an elevated risk of thrombosis, though its underlying molecular mechanisms remain incompletely understood. In this case-control study, involving 71 EM patients and 71 matched controls, we explored the structural and functional changes in fibrinogen and their potential role in thrombosis. Key oxidative stress markers, such as reactive oxygen species (ROS) levels in blood lymphocytes, monocytes, and granulocytes, along with plasma lipid peroxidation markers and total antioxidant capacity, were measured. Fibrinogen structure was examined using circular dichroism spectroscopy and intrinsic fluorescence, while functional properties were evaluated by analyzing thrombin-mediated polymerization and plasmin-induced lysis. Compared to controls, EM patients exhibited elevated ROS production and systemic oxidative stress, leading to notable fibrinogen oxidation and structural alterations. These changes were associated with impaired fibrin polymerization and enhanced resistance to plasmin-induced lysis, which are indicative of a pro-thrombotic state. These findings suggest that oxidative stress-driven fibrinogen modifications may contribute to the heightened thrombotic risk in women with EM, highlighting a potential therapeutic target to mitigate cardiovascular complications.

## 1. Introduction

Endometriosis (EM) is a common disorder of the female reproductive system, characterized by a chronic inflammatory process driven by estrogens [1,2,3,4]. A hallmark of this condition is the presence of endometrial tissue, including glands or stroma, outside the uterine cavity, particularly in the peritoneal cavity, the ovaries, ovarian fossa, uterosacral ligaments, and posterior cul-de-sac [5,6,7]. EM is a significant public health issue affecting approximately 176 million women worldwide, or about 5–10% of women of reproductive age [8,9]. It is the most common cause of chronic pelvic pain and is often associated with infertility [1,10,11]. In the development and progression of EM inflammation and oxidative stress represent key factors [12,13,14,15,16]. Increased reactive oxygen species (ROS) production can lead to cellular damage, DNA mutations, and the activation of pro-inflammatory pathways [17,18,19], which can perpetuate the inflammatory response in endometriotic lesions [20,21,22,23,24]. Inflammation and oxidative stress are interconnected, and each can exacerbate the other. For example, pro-inflammatory cytokines can stimulate the production of ROS, while ROS can activate inflammatory signaling pathways [25,26]. This cycle of inflammation and oxidative stress can lead to tissue damage and contribute to the chronic nature of EM [27].

EM has been linked to a higher risk of thrombotic events and cardiovascular diseases [28,29]. This association may stem from the chronic inflammation characteristic of EM, which can induce changes in the blood that increase its tendency to clot. Additionally, some studies have suggested that the hormonal changes associated with EM may also play a role in increasing the risk of thrombosis [30]. However, the underlying mechanisms that link EM and thrombosis have not been fully clarified.

Our research group has recently provided new insights into the relationship between ROS-mediated modifications of fibrinogen structure and its biological activity [31,32,33]. The data indicate that fibrinogen oxidation plays a role in the formation of more thrombogenic clots, characterized by a dense fibrin network made up of filaments that are resistant to plasmin-induced lysis, leading to a slight reduction in fiber size [34,35,36,37,38,39,40,41,42,43,44,45,46,47,48]. This extensively modified fibrin network may significantly contribute to vascular occlusion and thrombus formation [49,50,51,52,53,54,55].

On these bases, we investigated fibrinogen structural/functional modification and fibrin susceptibility to plasmin-induced lysis to gain a better understanding of the mechanisms underlying thrombus formation in EM.

## 2. Materials and Methods

### 2.1. Design of the Study

This cross-sectional case-control study was developed in accordance with the ethical standards reported in the 1964 Declaration of Helsinki and its later amendments [56] and was approved by the local Ethics Committee (Reference: 21140). All enrolled patients, before participating, signed informed consent forms. The study sample included 71 patients with stage III/IV of EM, defined according to the American Society for Reproductive Medicine score [57], and 71 age- and sex-matched healthy control subjects. All women were referred to the Internal Medicine Clinic at the Centre for Assisted Reproductive Technology, Division of Obstetrics and Gynecology of Careggi University Hospital, Florence. In all patients, the diagnosis of endometriosis was confirmed by diagnostic imaging (US or MRI) and/or laparoscopy performed by gynecologists expert in this field. Pre-existing atherothrombotic disorders, hypertension, diabetes, autoimmune diseases, renal failure, obesity, pregnancy, and inflammatory diseases represented exclusion criteria. Moreover, subjects with systemic vasculitis/autoimmune diseases, active infections, or neoplastic conditions were excluded from this study. Both the control group and women with endometriosis were undergoing therapy with either estrogen-progestins or progestins.

Demographic and clinical characteristics of the population studied are summarized in Table 1.

### 2.2. Sample Collection and Fibrinogen Purification

Blood samples were collected using BD Vacutainer blood collection tubes (Becton, Dickinson and Company, Franklin Lakes, NJ, USA) containing either trisodium citrate (1:10) or EDTA (0.17 mM). After centrifugation at 1500× *g* for 15 min at 4 °C, aliquots of sodium citrate plasma were used in experiments or stored at −80 °C for later analysis. Another aliquot of sodium citrate plasma underwent fibrinogen purification via ethanol precipitation, as described previously [58]. Fibrinogen concentration was measured using a UV/Vis spectrophotometer (ONDA UV-20, GEASS s.r.l., Torino, Italy) at 280 nm, with an assumed extinction coefficient of 1.51 mg/mL. EDTA blood samples were immediately utilized for fluorescence-activated cell sorting (FACS) analysis.

### 2.3. Measurement of ROS Production in Peripheral Leukocytes

The production of intracellular ROS in leukocytes was evaluated using previously established protocols [59,60,61]. Briefly, 100 μL of EDTA-anticoagulated blood samples were mixed with 2 mL of red blood cell lysis buffer (BioVision, Milpitas, CA, USA). The mixture was gently stirred and left at room temperature in the dark for 10 min, as per the manufacturer’s guidelines. After incubation, samples were centrifuged, supernatant discarded, and cells washed twice with phosphate-buffered saline (PBS). Leukocyte ROS production was measured by incubating cells with H_2_DCFDA (2.0 μmol/L; Invitrogen, Waltham, MA, USA) in serum-free, phenol-red-free RPMI medium (Merck KGaA, Darmstadt, Germany). Following labeling, the cells were washed, resuspended in PBS, and immediately analyzed using a FACSCanto flow cytometer (Becton, Dickinson and Company). The sample flow rate was adjusted to about 1000 cells/s. For a single analysis, the fluorescence properties of 5000 monocytes were collected. The respective gates were defined using the distinctive forward-scatter and side-scatter properties of the individual cell populations. Data analysis was performed with BD FACSDiva software v8.0.2 (Becton, Dickinson and Company, Franklin Lakes, NJ, USA).

### 2.4. Plasma Lipid Peroxidation Assay

Plasma lipid peroxidation was measured using an ALDetect Lipid Peroxidation assay (BML-AK170-Enzo Biochem Inc., New York, NY, USA), as previously reported [62].

### 2.5. Plasma Nitrate/Nitrite Content Assay

Plasma nitrate/nitrite concentration was determined using a spectrophotometric method, according to the manufacturer’s instructions (Cayman Chemical, Ann Arbor, MI, USA).

### 2.6. Determination of Plasma Total Antioxidant Capacity

The plasma total antioxidant capacity (TAC) was determined using the oxygen radical absorbance capacity (ORAC) assay. This method is based on the fluorescence decay of fluorescein due to oxidation by peroxyl radicals, which are produced from the thermal decomposition of azo compounds, such as 2,2′-azobis(2-amidinopropane) dihydrochloride. A solution of 6 nM fluorescein in 75 mM sodium phosphate buffer (pH 7.4) and 250 μM Trolox (Merck KGaA), a water-soluble vitamin E analog used as a standard, was utilized. After pre-incubating the samples at 37 °C for 30 min with 100 μL of fluorescein, a 19 mM final concentration of 2,2′-azobis(2-amidinopropane) dihydrochloride was added to initiate the reaction. Fluorescence was measured using a microplate fluorometer (BioTek Synergy H1, Agilent Technologies, Santa Clara, CA, USA), with excitation set at 485 nm and emission at 537 nm. The results were expressed in Trolox equivalents (nanomoles per milliliter) [63].

### 2.7. Fibrinogen Oxidation Assessment

Fibrinogen oxidation was evaluated by determining the dityrosine content in purified fibrinogen fractions, using a PerkinElmer LS 55 spectrofluorometer with a thermostated cell holder connected to a Haake F8 water bath, as described in a previous study [59].

### 2.8. Fibrinogen Structure Determination

The secondary structure of fibrinogen was analyzed using circular dichroism (CD) spectroscopy, with spectra recorded on a Jasco Fluorometer (Jasco 810, JASCO Europe Srl, Cremella, Italy). CD is a powerful technique for rapidly determining protein secondary structure, measuring the differential absorption of left- and right-handed circularly polarized light. When molecules interact with light, they may absorb these polarized light forms to varying degrees, which is the basis of CD. Different structural elements of the protein produce distinct CD spectra [64]. The spectra were collected at 25 °C using 0.2 cm quartz cells over a wavelength range of 280–180 nm (far UV) with a fibrinogen concentration of 0.5 mg/mL. Samples were filtered through 0.22 μM filters, and three spectra were recorded for each sample. Molar ellipticity values (θ) were calculated using the equation: [θ] (deg·cm^2^·dmol^−1^) = [θ (MRW)]/[10(l)(c)], where θ represents the shift from the baseline value, MRW is the mean residue weight of the amino acids, l is the cell path length (cm), and c is the protein concentration (g/mL).

Additionally, fibrinogen’s intrinsic fluorescence spectra were obtained to assess changes in its spatial conformation [59]. Protein intrinsic fluorescence is sensitive to aromatic amino acids such as phenylalanine, tyrosine, and tryptophan. Tryptophan fluorescence, in particular, is useful for monitoring structural changes, as its maximum emission is highly sensitive to the polarity of the surrounding environment. Both the intensity and wavelength of maximum emission for tryptophan fluorescence provide insights into the protein’s tertiary structure. The maximum intensity of fibrinogen intrinsic fluorescence (347 nm) was recorded at a protein concentration of 0.5 mg/mL at 25 °C in PBS using a PerkinElmer LS 55 spectrofluorometer (PerkinElmer Inc., Waltham, MA, USA). This setup included a thermostated cell holder connected to a Haake F8 water bath, and an excitation wavelength of 280 nm was used with a 2 mm × 10 mm quartz cuvette.

### 2.9. Fibrinogen Functional Analysis

The functional analysis of fibrinogen included assessing the kinetics of thrombin-catalyzed fibrin polymerization and the susceptibility of fibrin to plasmin-induced lysis. Thrombin-catalyzed fibrin polymerization was initiated and monitored as described previously [59]. The resulting absorbance curves were analyzed based on the following parameters: (i) Maximum slope (Vmax), representing the steepest part of the curve. (ii) Lag phase, defined as the time (in minutes) before an increase in absorbance was detected. (iii) Maximum absorbance (Max Abs) of the clot, recorded 120 min after the start of polymerization.

Additionally, fibrin digestion by plasmin was monitored following previously established methods [59]. Fibrin clots were formed in microcentrifuge tubes by incubating human thrombin (final concentration 12 units/mL) with 10 μg fibrinogen in 20 μL of 100 mM Tris/HCl, 5 mM CaCl_2_, pH 7.4 for 2 h at 25 °C. Afterward, plasmin (5 μL of 100 μg/mL) was added to the clots, which were digested over a 6 h period at 37 °C. The reaction was stopped by adding 10 μL of lithium dodecyl sulfate gel electrophoresis sample buffer. The same lot of thrombin and plasmin was used in all experiments. Samples were heated at 70 °C for 10 min under reducing conditions (50 mM dithiothreitol). Aliquots equivalent to 10 μg of fibrin from each digest were then loaded onto 4% to 12% Bis-Tris gels. Following electrophoresis, gels were stained with Coomassie blue, and band intensities were quantified by densitometry using the ChemiDoc system and Quantity-One 4.6 software (Bio-Rad Laboratories, Hercules, CA, USA). The results were expressed as a percentage of the densitometric reading of the fibrin beta chain after 6 h of plasmin digestion relative to the beta band reading of undigested fibrin at time 0. A higher value indicates lower fibrin degradation (and potentially a higher cardiovascular risk).

### 2.10. Statistical Analysis

Categorical variables were reported as absolute frequencies and percentages, and compared between EM patients and controls using the Fisher exact test. Continuous variables were described as median values and interquartile range (IQR), and were compared between EM patients and controls using the Mann–Whitney test for unpaired data. Data distribution was assessed with the Shapiro–Wilk test. All experiments were performed in triplicate and, for each subject, the mean of the three experiments was used, after verifying low intra-experiment and inter-experiment variability and reproducibility of measures using the ANOVA Bonferroni Test. Correlations between oxidative stress biomarkers and fibrinogen structural and functional parameters were analyzed using Spearman’s rank correlation test.

## 3. Results

### 3.1. Intracellular Leukocyte ROS Production

**Figure 1:**
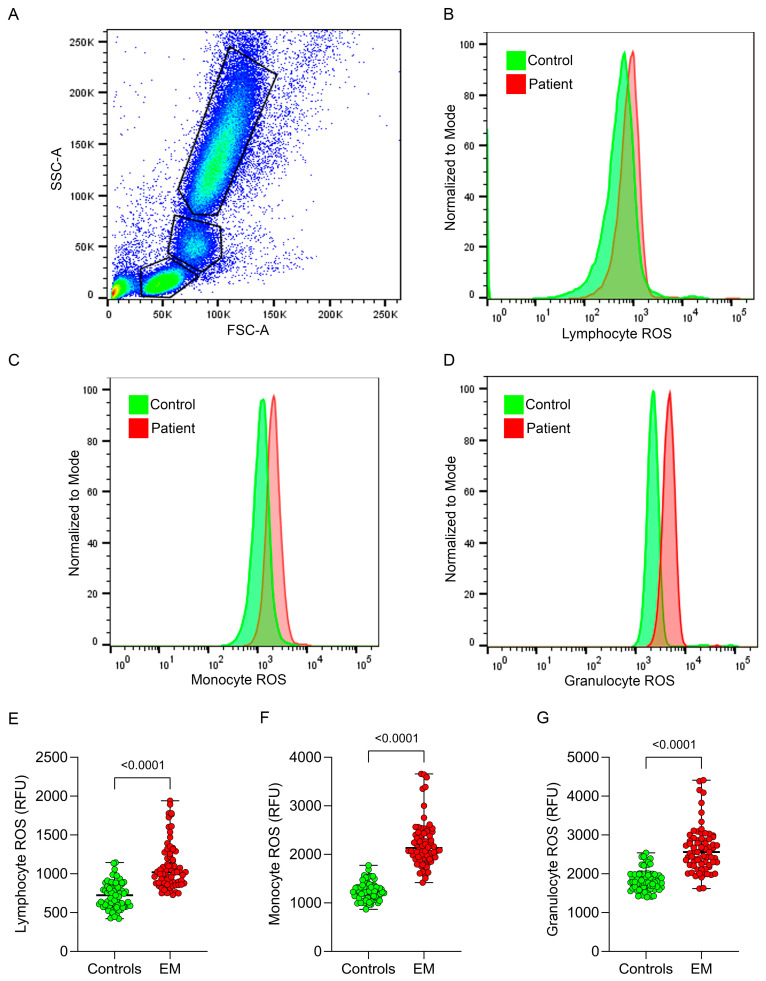
Intracellular leukocyte ROS production by FACS analysis. (**A**) Representative gating strategy for identifying leukocyte subpopulations based on forward scatter (FSC-A) and side scatter (SSC-A) properties. Regions were defined to distinguish lymphocytes, monocytes, and granulocytes. (**B**–**D**) Histogram overlays showing ROS levels (measured as relative fluorescence units, RFUs) in lymphocytes (**B**), monocytes (**C**), and granulocytes (**D**) from endometriosis (EM) patients (red) and controls (green). (**E**–**G**) Quantification of ROS levels in lymphocytes (**E**), monocytes (**F**), and granulocytes (**G**) from controls and EM patients, expressed as relative fluorescence units (RFUs).

In Figure 1, intracellular ROS production in blood leukocytes from EM patients and matched controls is shown. ROS levels across all three leukocyte fractions in EM patients were significantly increased compared to controls (Figure 1B–D). Specifically, lymphocyte ROS were 1020 (879–1255) RFU in EM patients versus 723 (578–856) RFU in controls (*p* < 0.0001; Figure 1E), monocyte ROS levels were 2132 (1922–2431) RFU versus 1222 (1105–1348) RFU (*p* < 0.0001; Figure 1F), and granulocyte ROS levels were 2561 (2225–2908) RFU versus 1789 (1660–1996) RFU in controls (*p* < 0.0001; Figure 1G). These results indicate that oxidative stress is elevated in leukocytes from EM patients.

### 3.2. Systemic Redox Status

**Figure 2:**
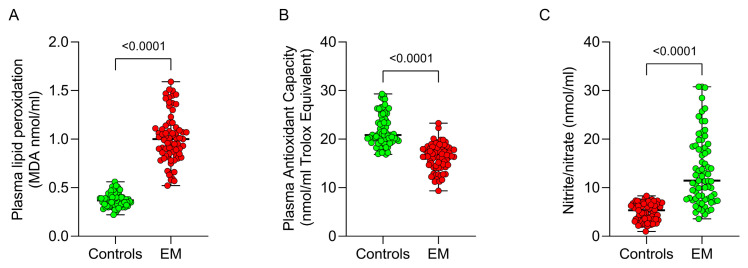
Plasma oxidative stress markers in EM patients. MDA (**A**), total antioxidant capacity (**B**) and nitrate/nitrite levels (**C**) in EM patients and controls.

Redox status in plasma was assessed by measuring MDA (malondialdehyde, the main lipid peroxidation marker), total antioxidant capacity (TAC), and nitrate/nitrite levels. Lipid peroxidation was significantly higher in EM patients compared to controls (1.00 [0.83–1.12] vs. 0.37 [0.32–0.41] MDA nmol/mL; *p* < 0.0001), along with a marked reduction in TAC (16.41 [14.37–17.95] vs. 20.86 [19.66–24.22] mM Trolox equivalent; *p* < 0.0001; Figure 2A,B). Additionally, EM patients exhibited a significant increase in plasma nitrate/nitrite levels (11.46 [7.76–18.48] vs. 5.34 [3.5–6.86] nmol/mL; *p* < 0.0001; Figure 2C). These findings suggest systemic oxidative stress in EM patients.

### 3.3. Fibrinogen Structural Changes and Fibrinogen Oxidation

**Figure 3:**
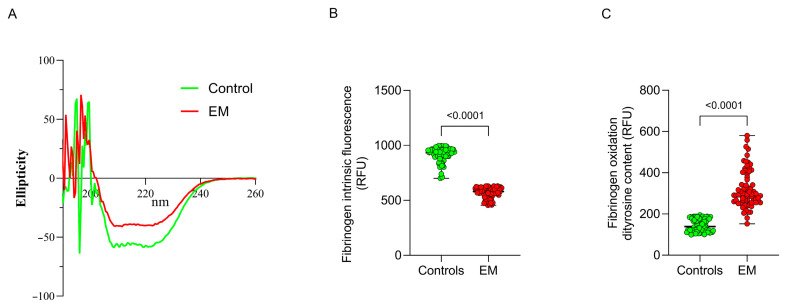
Fibrinogen structural changes and oxidation in EM patients and controls. Representative fibrinogen circular dichroism spectra (fibrinogen secondary structure, (**A**)), intrinsic fibrinogen fluorescence (fibrinogen tertiary structure, (**B**)) and fibrinogen dityrosine content (fibrinogen oxidation, (**C**)) in EM patients and controls.

The protein function is closely linked to its structure and even minor changes in protein structure can significantly affect its biological activity. To evaluate the effect of fibrinogen oxidation on its structure, the far-UV CD spectra of fibrinogen purified from EM patients and matched controls were qualitatively compared. In controls, fibrinogen revealed a typical α-helical secondary structure with characteristic minima at 208 and 222 nm. In contrast, a decreased negative peak in the 215–225 nm region, indicating a reduced α-helical content (Figure 3A), and hence structural alterations, were found in fibrinogen from EM patients. The α-helix secondary structure is essential for maintaining fibrinogen’s structural integrity and functional properties. Rich in oxidation-prone amino acids like methionine, cysteine, and tyrosine, these α-helix regions are particularly vulnerable to modifications such as carbonylation, dityrosine formation, and disulfide bond alterations under oxidative stress. Such disruptions destabilize the protein structure, impairing fibrinogen’s ability to polymerize into fibrin and form clots with normal mechanical properties.

Furthermore, we assayed, in the purified fibrinogen samples, the intrinsic fluorescence which reflects the exposure of hydrophobic amino acid residues to the solvent and the protein tertiary structure. Significant differences in fibrinogen fluorescence intensity were observed between EM patients and controls (580 [530–608] vs. 934 [900–964] RFU, *p* < 0.0001; Figure 3B), indicating structural alterations.

To explore the relationship between systemic oxidative stress and fibrinogen oxidation, dityrosine content in purified fibrinogen was assessed. Dityrosine content is a precise indicator of protein oxidation, formed through covalent cross-linking between tyrosine residues during oxidative stress. Increased levels of ROS drive oxidative modifications in proteins, rendering tyrosine residues susceptible to dityrosine bond formation. This cross-linking reflects the extent of oxidative damage while also impacting the protein’s structure and function. Dityrosine content was significantly increased in EM patients compared to controls (293 [260–378] vs 139 [119–176] RFU, *p* < 0.0001; Figure 3C). These findings suggest that fibrinogen in EM patients is structurally altered, likely due to increased oxidation.

### 3.4. Fibrinogen Functional Assessment

**Figure 4:**
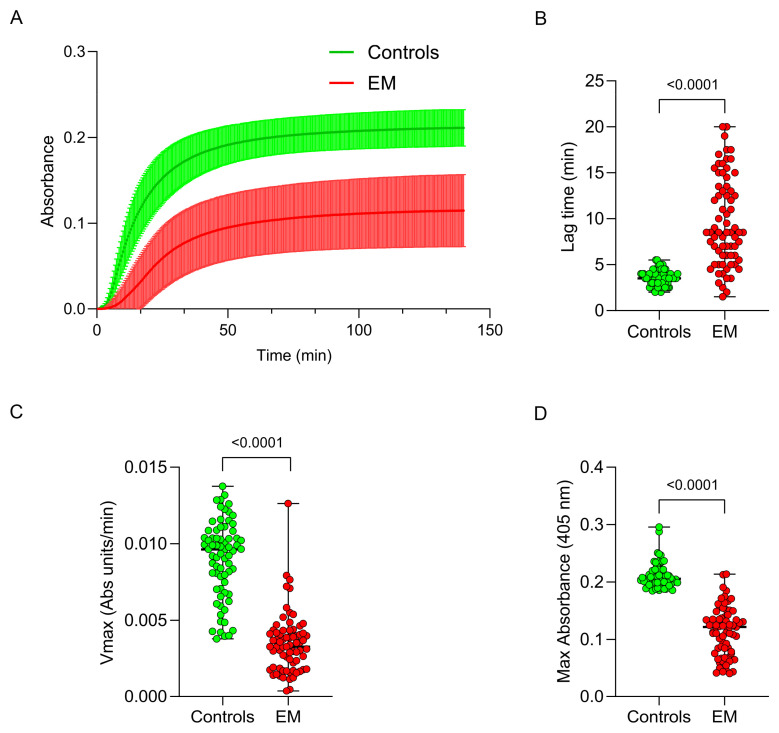
Fibrinogen functional modifications in EM patients compared to controls. Fibrinogen polymerization into fibrin in EM patients and controls is shown in Panel (**A**). Lag phase, maximum slope (Vmax), and maximum absorbance (Max abs) of fibrinogen polymerization curves in EM patients and controls are shown in the panels (**B**–**D**). The observed modifications are related to a different fibrinogen structure in EM fibrinogen compared to controls.

The impact of fibrinogen structural alterations on its function in EM patients and matched controls was assessed by analyzing thrombin-catalyzed fibrin polymerization and fibrin susceptibility to plasmin-induced lysis. A diminished capacity to polymerize into fibrin was evident in fibrinogen from EM patients (Figure 4A), as witnessed by significant differences in key polymerization parameters—lag phase, maximum velocity (Vmax), and maximum absorbance (Max Abs)—between EM patients and controls. Specifically, EM patients showed a prolonged lag phase (8.5 [6.00–13.50] vs. 3.50 [3.00–4.00], *p* < 0.0001), along with a reduced Vmax (0.0033 [0.0019–0.0042] vs. 0.0096 [0.0071–0.0108], *p* < 0.0001) and Max Abs (0.122 [0.079–0.139] vs. 0.201 [0.197–0.222], *p* < 0.0001; Figure 4B–D).

**Figure 5:**
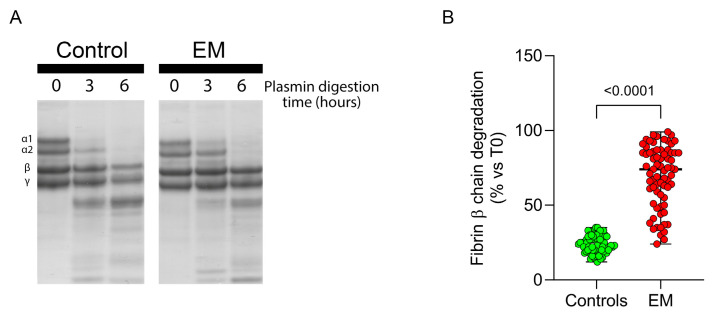
EM patients show fibrin resistance to plasmin-induced lysis. Representative gel showing fibrin degradation at 0 h, 3 h, and 6 h of plasmin digestion, with fibrinogen purified from EM patients and controls (**A**). Fibrin β chain degradation rates (**B**) in EM patients and controls.

Additionally, when assessing fibrin susceptibility to plasmin-induced lysis, evaluated by quantifying the degradation of the fibrin β chain over 0 to 6 h of plasmin digestion (Figure 5A), fibrin from EM patients showed significantly higher levels of undigested fibrin compared to controls (74 [55–85] vs. 23 [20–29]; *p* < 0.0001), indicating increased resistance to fibrinolysis in EM patients (Figure 5B).

### 3.5. Correlation Between Redox Status and Fibrinogen Alterations

In EM patients, the correlation between fibrinogen oxidation and changes in fibrinogen structure/function was investigated (Table 2). Our findings indicate a correlation between fibrin degradation and granulocyte ROS production, as well as markers of fibrinogen oxidation (dityrosine content) and fibrinogen structural alterations (fibrinogen intrinsic fluorescence). Additionally, leukocyte ROS production and the plasma markers of redox status also exhibited statistically significant correlations, as indicated below.

## 4. Discussion

Endometriosis is a chronic inflammatory gynecological condition characterized by inflammation, pain, and infertility. Its development results from a complex interaction of genetic, epigenetic, immunological, and environmental factors. Recent studies have shown that women with endometriosis are at a heightened risk of thrombotic events [65,66,67,68,69,70], likely due to the chronic inflammation that defines the disease [4,71,72]. Additionally, these women may exhibit other risk factors for thrombosis, including hormonal imbalances and sedentary lifestyles. However, the precise mechanisms linking endometriosis and thrombosis remain unclear, necessitating further investigation. Recent evidence highlights the role of redox imbalance in macrophage hyperactivation, which contributes to erythrocyte degradation and the release of pro-oxidant and pro-inflammatory factors, promoting ROS formation at the pelvic level [73,74]. It is well established that the coagulation system is intricately linked to inflammation, with inflammatory processes driving coagulation and elevating thrombosis risk [75]. Studies have demonstrated a hypercoagulative and thrombotic tendency in women with endometriosis, reflected by increased fibrinogen levels [76,77]. Furthermore, immune cells in these patients release cytokines and defensins, exacerbating the inflammatory environment [78]. Despite these findings, the molecular mechanisms connecting endometriosis and thrombosis remain poorly understood.

In this study, we examined whether oxidative-induced structural and functional changes in fibrinogen contribute to thrombosis in patients with endometriosis.

Our results suggest a significant systemic redox imbalance in EM patients, strongly associated with increased fibrinogen oxidation. Plasma redox imbalance in endometriosis was confirmed by the decreased total antioxidant capacity (TAC) and elevated malondialdehyde (MDA) levels compared to the controls, consistent with prior studies documenting oxidative stress in endometriosis [79,80,81]. Amreen et al. similarly reported a significant correlation between lipid peroxidation levels in blood and peritoneal fluid and the severity of endometriosis [81]. Elevated plasma nitrite/nitrate levels further confirm systemic oxidative stress in endometriosis patients. Oxidative processes are known to alter protein conformation, potentially generating (auto)antigens that can trigger inflammation, tissue damage, immune dysregulation, and thrombosis [31,82,83]. Our findings reveal a strong association between fibrinogen oxidation and structural and functional changes in the protein. For the first time, far-UV circular dichroism spectroscopy demonstrated alterations in the secondary structure of fibrinogen in EM patients, with a reduced α-helix-rich regions. Additionally, intrinsic fluorescence analysis revealed extensive modifications to the tertiary fibrinogen, likely due to the exposure of hydrophobic amino acids to the surrounding environment. These structural changes significantly impact fibrin polymerization and increase susceptibility to plasmin-induced lysis [44,46,47,84,85]. Reduced plasma clot permeability and prolonged clot lysis time observed in endometriosis patients align with our findings and suggest denser, fibrinolysis-resistant plasma fibrin clots [86].

Our results also showed that fibrin formation was significantly altered in EM patients, corroborating earlier in vitro findings that fibrinogen carbonylation impairs clotting capacity [87]. The presence of the oxidation-prone amino acid arginine at fibrinogen’s thrombin-cleavage site may partially explain the altered polymerization kinetics seen in EM patients [46]. Reduced Vmax and final turbidity underscore the structural differences in fibrinogen and its interaction with thrombin. In vitro and ex vivo studies, including ours, consistently report prolonged lag phases and reduced maximum absorbance and velocity in the turbidity assays of oxidized fibrinogen [31,32,44,82,87,88,89,90,91,92,93,94,95,96,97]. However, some studies note increased polymerization rates under specific conditions [97,98].

This study has several limitations. First, we did not investigate a direct link between fibrinogen oxidative changes and thrombotic events in endometriosis. Future prospective studies with larger sample sizes and extended follow-up periods are necessary to address this gap. Second, we did not analyze genetic polymorphisms influencing fibrinogen structure, nor did we investigate γ′ fibrinogen, which results from alternative mRNA processing and produces fibrinolysis-resistant thrombi [99]. Although systemic oxidative stress in EM patients was evident, we cannot conclusively establish a direct causal relationship between fibrinogen alterations and redox imbalance based solely on these findings. However, our previous data support the notion that leukocyte ROS production and systemic oxidative stress can be involved in fibrinogen alteration, leading to prothrombotic features [31,59,87]. Moreover, hormonal therapy, may influence oxidative stress and fibrinogen modifications, potentially affecting thrombotic risk [86,100,101,102]. Estrogen can both increase and mitigate oxidative stress depending on dosage, while progestins generally have a neutral or anti-inflammatory impact [103,104,105,106,107]. These therapies can modulate fibrin structure and fibrinolysis resistance differently, potentially amplifying or reducing pro-thrombotic tendencies. However, the pronounced differences in oxidative stress and fibrinogen alterations between endometriosis patients and controls, despite both groups being on hormonal therapy, suggest that the pro-thrombotic state is primarily driven by the disease itself rather than the treatment. The classification of all patients as stage III/IV endometriosis underscores the link between advanced disease severity and heightened inflammation. These stages, marked by extensive adhesions and deep infiltrating lesions, are associated with increased pro-inflammatory cytokines (e.g., IL-6, TNF-α) and oxidative stress, driving chronic tissue damage and systemic inflammation [68,108,109]. Our findings of elevated oxidative stress and fibrinogen modifications align with the advanced inflammatory burden in these stages, which may also contribute to greater thrombotic risk. These results highlight the importance of considering disease stage in evaluating inflammation and thrombotic risk in endometriosis.

In conclusion, this study is the first to purify fibrinogen from women with endometriosis and conduct a comprehensive evaluation of its structural and functional properties. By analyzing fibrinogen’s structural alterations, we provide novel insights into the pro-thrombotic state in EM patients, offering a partial explanation for their increased thrombotic risk. Our results underscore, for the first time, significant structural changes in fibrinogen, including altered polymerization kinetics, reduced susceptibility to plasmin-induced lysis, and systemic redox imbalance. These findings highlight the critical role of oxidative stress in fibrinogen modifications and the associated thrombotic risk.

Further research is required to explore direct causal links between fibrinogen alterations and thrombotic events, and to develop novel diagnostic tools for assessing cardiovascular risk in endometriosis patients. Additionally, our findings emphasize the potential for innovative therapeutic strategies aimed at restoring redox balance. Such approaches could improve the management of the prothrombotic state in endometriosis, potentially reducing cardiovascular complications and offering more personalized and effective treatments.

## Figures and Tables

**Table 1:** Baseline characteristics of the study population.

Variables	Patients (*n* = 71)	Controls (*n* = 71)	*p* Value
**Age, y, median (range)**	30 (20–47)	32 (20–39)	0.4049
**Weight, kg, median (range)**	58 (44–87)	56.5 (45–78)	0.3470
**BMI, kg/m^2^, median (range)**	21 (16.2–29.5)	22.5 (16.1–28.6)	0.9246
**Underweight (<18.5 kg/m^2^), *n* (%)**	17 (23.9)	10 (14.1)	0.1989
**Overweight (25–29.99 kg/m^2^), *n* (%)**	9 (12.7)	6 (8.5)	0.5864
**Waist circumference ≥ 80 cm, *n* (%)**	17 (23.9)	N/A	-
**WHR ≥ 0.85, *n* (%)**	11 (15.5)	N/A	-
**Smoking habit, *n* (%)**	18 (23.4)	24 (33.8)	0.3580
**Migraine, *n* (%)**	31 (43.7)	N/A	-
**Migraine with aura, *n* (%)**	7 (9.9)	N/A	-
**Dyslipidaemia, *n* (%)**	36 (50.7)	39 (54.9)	0.7369
**Total cholesterol > 200 mg/dL, *n* (%)**	22 (31)	24 (33.8)	0.8578
**LDL cholesterol > 116 mg/dL, *n* (%)**	29 (40.8)	31 (43.66)	0.8652
**HDL cholesterol < 48 mg/dL, *n* (%)**	7 (9.9)	6 (8.5)	1
**Triglycerides > 150 mg/dL, *n* (%)**	3 (4.2)	4 (5.6)	1
**Lipoprotein (a) > 300 mg/L, *n* (%)**	18 (23.4)	N/A	-
**History of negative obstetric events, *n* (%)**	2 (2.8)	N/A	-
**Family history of endometriosis, *n* (%)**	14 (19.7)	N/A	-
**Family history of CVD, *n* (%)**	19 (26.7)	N/A	-
**Fibrinogen mg/dL, median (range)**	278 (150–550)	322 (157–411)	0.5702

BMI indicates body mass index; WHR, waist-to-hip ratio; LDL, low-density lipoprotein; HDL, high-density lipoprotein; CVD, cardiovascular disease; N/A, not available.

**Table 2:** Correlation analysis.

	ROS Limpho	ROS Mono	ROS Granu	MDA	ORAC	Nitrate/ Nitrite	Fibrin Deg	Max Abs	Lag Time	Vmax	Dityr	IF
**ROS Lympho**		0.89 *	0.80 *	0.184	−0.020	0.238 *	0.100	−0.053	0.113	−0.035	−0.026	0.033
**ROS Mono**	0.89 *		0.85 *	0.122	−0.012	0.176	0.134	−0.057	0.088	0.068	0.003	−0.008
**ROS Granu**	0.80 *	0.85 *		0.092	0.117	0.210	0.370	−0.058	0.030	−0.027	0.049	−0.007
**MDA**	0.184	0.122	0.092		−0.051	0.268 *	−0.177	0.051	0.021	−0.054	−0.13	0.015
**ORAC**	−0.020	−0.012	0.117	−0.051		0.182	0.101	−0.143	−0.150	0.107	0.006	−0.085
**Nitrate/Nitrite**	0.238 *	0.176	0.210	0.268 *	0.182		0.196	−0.031	−0.137	−0.009	0.173	−0.324 *
**Fibrin Deg**	0.100	0.134	0.37 *	−0.177	0.101	0.196		0.144	−0.045	0.226	0.060	−0.26 *
**Max Abs**	−0.053	−0.057	−0.058	0.051	−0.143	−0.031	0.144		0.211	0.26 *	−0.006	0.151
**Lag Time**	0.113	0.088	0.030	0.021	−0.150	−0.137	−0.045	0.211		−0.128	0.097	−0.023
**Vmax**	−0.035	0.068	−0.027	−0.054	0.107	−0.009	0.226	0.26 *	−0.128		0.100	−0.198
**Dityr**	−0.026	0.003	0.049	−0.13	0.006	0.173	0.060	−0.006	0.097	0.100		−0.42 *
**IF**	0.033	−0.008	−0.007	0.015	−0.085	−0.324 *	−0.26 *	0.151	−0.023	−0.198	−0.42 *	

Dityr, dityrosine content; Fibrin Deg, fibrin degradation; IF, intrinsic fluorescence; Lympho, lymphocytes; Max Abs, maximum absorbance; Mono, monocytes; Granu, granulocytes; ORAC, oxygen radical absorbance capacity; Vmax, maximum slope. The correlation matrix displays Spearman’s R values; correlations with a *p*-value < 0.05 are indicated with the symbol *.

## Data Availability

All data relevant to the study are included in the article, further inquiries can be directed to the corresponding author.

## References

[1] Bulun S.E., Yilmaz B.D., Sison C., Miyazaki K., Bernardi L., Liu S., Kohlmeier A., Yin P., Milad M., Wei J. (2019). Endometriosis. Endocr. Rev..

[2] Smolarz B., Szyłło K., Romanowicz H. (2021). Endometriosis: Epidemiology, Classification, Pathogenesis, Treatment and Genetics (Review of Literature). Int. J. Mol. Sci..

[3] Kobayashi H., Imanaka S., Yoshimoto C., Matsubara S., Shigetomi H. (2024). Rethinking the pathogenesis of endometriosis: Complex interactions of genomic, epigenetic, and environmental factors. J. Obs. Gynaecol. Res..

[4] Rathod S., Shanoo A., Acharya N. (2024). Endometriosis: A Comprehensive Exploration of Inflammatory Mechanisms and Fertility Implications. Cureus.

[5] Giudice L.C., Kao L.C. (2004). Endometriosis. Lancet.

[6] Wang Y., Nicholes K., Shih I.M. (2020). The Origin and Pathogenesis of Endometriosis. Annu. Rev. Pathol..

[7] Vercellini P., Viganò P., Somigliana E., Fedele L. (2014). Endometriosis: Pathogenesis and treatment. Nat. Rev. Endocrinol..

[8] Halme J., Hammond M.G., Hulka J.F., Raj S.G., Talbert L.M. (1984). Retrograde menstruation in healthy women and in patients with endometriosis. Obs. Gynecol..

[9] Mehedintu C., Plotogea M.N., Ionescu S., Antonovici M. (2014). Endometriosis still a challenge. J. Med. Life.

[10] The Practice Committee of the American Society for Reproductive Medicine (2006). Endometriosis and infertility. Fertil. Steril..

[11] Trinchant R., García-Velasco J.A. (2024). Oocyte Quality in Women with Endometriosis. Gynecol. Obs. Investig..

[12] Augoulea A., Alexandrou A., Creatsa M., Vrachnis N., Lambrinoudaki I. (2012). Pathogenesis of endometriosis: The role of genetics, inflammation and oxidative stress. Arch. Gynecol. Obs..

[13] Samimi M., Pourhanifeh M.H., Mehdizadehkashi A., Eftekhar T., Asemi Z. (2019). The role of inflammation, oxidative stress, angiogenesis, and apoptosis in the pathophysiology of endometriosis: Basic science and new insights based on gene expression. J. Cell. Physiol..

[14] Cirillo M., Argento F.R., Attanasio M., Becatti M., Ladisa I., Fiorillo C., Coccia M.E., Fatini C. (2023). Atherosclerosis and Endometriosis: The Role of Diet and Oxidative Stress in a Gender-Specific Disorder. Biomedicines.

[15] Cuffaro F., Russo E., Amedei A. (2024). Endometriosis, Pain, and Related Psychological Disorders: Unveiling the Interplay among the Microbiome, Inflammation, and Oxidative Stress as a Common Thread. Int. J. Mol. Sci..

[16] Ansariniya H., Yavari A., Javaheri A., Zare F. (2022). Oxidative stress-related effects on various aspects of endometriosis. Am. J. Reprod. Immunol..

[17] Bettiol A., Galora S., Argento F.R., Fini E., Emmi G., Mattioli I., Bagni G., Fiorillo C., Becatti M. (2022). Erythrocyte oxidative stress and thrombosis. Expert. Rev. Mol. Med..

[18] Becatti M., Mannucci A., Barygina V., Mascherini G., Emmi G., Silvestri E., Wright D., Taddei N., Galanti G., Fiorillo C. (2017). Redox status alterations during the competitive season in élite soccer players: Focus on peripheral leukocyte-derived ROS. Intern. Emerg. Med..

[19] Fiorillo C., Becatti M., Attanasio M., Lucarini L., Nassi N., Evangelisti L., Porciani M.C., Nassi P., Gensini G.F., Abbate R. (2010). Evidence for oxidative stress in plasma of patients with Marfan syndrome. Int. J. Cardiol..

[20] Wang X., Jiang X., Lv X., Lin A., Li Y. (2024). NADPH oxidase 4-mediating oxidative stress contributes to endometriosis. J. Appl. Genet..

[21] Didziokaite G., Biliute G., Gudaite J., Kvedariene V. (2023). Oxidative Stress as a Potential Underlying Cause of Minimal and Mild Endometriosis-Related Infertility. Int. J. Mol. Sci..

[22] Luo X., Wen S., Zeng J., Liu J., Ye W., Wu J., Huang S., Xie W., Wen H., Sun Y. (2024). AOPPs induces EMT and fibrosis by activating oxidative stress through ERK/p38 MAPK signaling pathway in endometriosis. Reprod. Biol..

[23] Ding Y., Luo Y., Fu J. (2014). Effects of Mn (II) on peroxynitrite nitrifying fibrinogen. Biomed. Mater. Eng..

[24] Arangia A., Marino Y., Fusco R., Siracusa R., Cordaro M., D’Amico R., Macrì F., Raffone E., Impellizzeri D., Cuzzocrea S. (2023). Fisetin, a Natural Polyphenol, Ameliorates Endometriosis Modulating Mast Cells Derived NLRP-3 Inflammasome Pathway and Oxidative Stress. Int. J. Mol. Sci..

[25] Emmi G., Becatti M., Bettiol A., Hatemi G., Prisco D., Fiorillo C. (2019). Behçet’s Syndrome as a Model of Thrombo-Inflammation: The Role of Neutrophils. Front. Immunol..

[26] Hong C., Li X., Zhang K., Huang Q., Li B., Xin H., Hu B., Meng F., Zhu X., Tang D. (2024). Novel perspectives on autophagy-oxidative stress-inflammation axis in the orchestration of adipogenesis. Front. Endocrinol..

[27] Nanda A.K.T., Banerjee P., Dutta M., Wangdi T., Sharma P., Chaudhury K., Jana S.K. (2020). Cytokines, Angiogenesis, and Extracellular Matrix Degradation are Augmented by Oxidative Stress in Endometriosis. Ann. Lab. Med..

[28] Mamillapalli R., Taylor H.S. (2022). Endometriosis causes cardiovascular disease. Am. J. Obs. Gynecol..

[29] Poeta do Couto C., Policiano C., Pinto F.J., Brito D., Caldeira D. (2023). Endometriosis and cardiovascular disease: A systematic review and meta-analysis. Maturitas.

[30] Machin N., Ragni M.V. (2020). Hormones and thrombosis: Risk across the reproductive years and beyond. Transl. Res..

[31] Bettiol A., Argento F.R., Fini E., Bello F., Di Scala G., Taddei N., Emmi G., Prisco D., Becatti M., Fiorillo C. (2023). ROS-driven structural and functional fibrinogen modifications are reverted by interleukin-6 inhibition in Giant Cell Arteritis. Thromb. Res..

[32] Gitto S., Fiorillo C., Argento F.R., Fini E., Borghi S., Falcini M., Roccarina D., La Delfa R., Lillo L., Zurli T. (2024). Oxidative stress-induced fibrinogen modifications in liver transplant recipients: Unraveling a novel potential mechanism for cardiovascular risk. Res. Pract. Thromb. Haemost..

[33] Becatti M., Mannucci A., Argento F.R., Gitto S., Vizzutti F., Marra F., Taddei N., Fiorillo C., Laffi G. (2020). Super-Resolution Microscopy Reveals an Altered Fibrin Network in Cirrhosis: The Key Role of Oxidative Stress in Fibrinogen Structural Modifications. Antioxidants.

[34] Undas A. (2014). Fibrin clot properties and their modulation in thrombotic disorders. Thromb. Haemost..

[35] Undas A., Zawilska K., Ciesla-Dul M., Lehmann-Kopydłowska A., Skubiszak A., Ciepłuch K., Tracz W. (2009). Altered fibrin clot structure/function in patients with idiopathic venous thromboembolism and in their relatives. Blood.

[36] Natorska J., Ząbczyk M., Mastalerz L., Undas A. (2024). Increased factor XI but not factor XII is associated with enhanced inflammation and impaired fibrin clot properties in patients with eosinophilic granulomatosis with polyangiitis. Clin. Exp. Rheumatol..

[37] Varjú I., Sorvillo N., Cherpokova D., Farkas Á., Farkas V.J., Komorowicz E., Feller T., Kiss B., Kellermayer M.Z., Szabó L. (2021). Citrullinated Fibrinogen Renders Clots Mechanically Less Stable, but Lysis-Resistant. Circ. Res..

[38] Varjú I., Tóth E., Farkas Á., Farkas V.J., Komorowicz E., Feller T., Kiss B., Kellermayer M.Z., Szabó L., Wacha A. (2022). Citrullinated fibrinogen forms densely packed clots with decreased permeability. J. Thromb. Haemost..

[39] Vilar R., Fish R.J., Casini A., Neerman-Arbez M. (2020). Fibrin(ogen) in human disease: Both friend and foe. Haematologica.

[40] Wang L., Li L., Wang H., Liu J. (2016). Study on the influence of oxidative stress on the fibrillization of fibrinogen. Biochem. J..

[41] Weigandt K.M., White N., Chung D., Ellingson E., Wang Y., Fu X., Pozzo D.C. (2012). Fibrin clot structure and mechanics associated with specific oxidation of methionine residues in fibrinogen. Biophys. J..

[42] Weisel J.W., Litvinov R.I. (2013). Mechanisms of fibrin polymerization and clinical implications. Blood.

[43] White N.J., Wang Y., Fu X., Cardenas J.C., Martin E.J., Brophy D.F., Wade C.E., Wang X., St John A.E., Lim E.B. (2016). Post-translational oxidative modification of fibrinogen is associated with coagulopathy after traumatic injury. Free. Radic. Biol. Med..

[44] Yurina L.V., Vasilyeva A.D., Bugrova A.E., Indeykina M.I., Kononikhin A.S., Nikolaev E.N., Rosenfeld M.A. (2019). Hypochlorite-Induced Oxidative Modification of Fibrinogen. Dokl. Biochem. Biophys..

[45] Yurina L., Vasilyeva A., Indeykina M., Bugrova A., Biryukova M., Kononikhin A., Nikolaev E., Rosenfeld M. (2019). Ozone-induced damage of fibrinogen molecules: Identification of oxidation sites by high-resolution mass spectrometry. Free. Radic. Res..

[46] Yurina L.V., Vasilyeva A.D., Vasserman L.A., Podoplelova N.A., Panteleev M.A., Rosenfeld M.A. (2021). Effect of Hypochlorite- and Peroxide-Induced Oxidation of Fibrinogen on the Fibrin Structure. Dokl. Biochem. Biophys..

[47] Yurina L.V., Vasilyeva A.D., Gavrilina E.S., Ivanov V.S., Obydennyi S.I., Chabin I.A., Indeykina M.I., Kononikhin A.S., Nikolaev E.N., Rosenfeld M.A. (2024). A role of methionines in the functioning of oxidatively modified fibrinogen. Biochim. Biophys. Acta Proteins Proteom..

[48] Ząbczyk M., Ariëns R.A.S., Undas A. (2023). Fibrin clot properties in cardiovascular disease: From basic mechanisms to clinical practice. Cardiovasc. Res..

[49] Tenopoulou M. (2024). Fibrinogen post-translational modifications are biochemical determinants of fibrin clot properties and interactions. FEBS J..

[50] Słaboszewski M., Kolec R., Paszek E., Baran M., Undas A. (2024). Prothrombotic plasma fibrin clot phenotype is associated with spontaneous echo contrast in atrial fibrillation: The role of protein carbonylation. Thromb. Res..

[51] Konieczyńska M., Natorska J., Undas A. (2024). Thrombosis and Aging: Fibrin Clot Properties and Oxidative Stress. Antioxid. Redox Signal..

[52] Sproul E.P., Hannan R.T., Brown A.C. (2018). Controlling Fibrin Network Morphology, Polymerization, and Degradation Dynamics in Fibrin Gels for Promoting Tissue Repair. Protocol.

[53] Azizova O.A., Piryazev A.P., Aseychev A.V., Shvachko A.G. (2009). Oxidative modification of fibrinogen inhibits its transformation into fibrin under the effect of thrombin. Bull. Exp. Biol. Med..

[54] de Vries J.J., Snoek C.J.M., Rijken D.C., de Maat M.P.M. (2020). Effects of Post-Translational Modifications of Fibrinogen on Clot Formation, Clot Structure, and Fibrinolysis: A Systematic Review. Arter. Thromb. Vasc. Biol..

[55] Schuett K., Savvaidis A., Maxeiner S., Lysaja K., Jankowski V., Schirmer S.H., Dimkovic N., Boor P., Kaesler N., Dekker F.W. (2017). Clot Structure: A Potent Mortality Risk Factor in Patients on Hemodialysis. J. Am. Soc. Nephrol..

[56] Goodyear M.D., Krleza-Jeric K., Lemmens T. (2007). The Declaration of Helsinki. BMJ.

[57] Haas D., Shebl O., Shamiyeh A., Oppelt P. (2013). The rASRM score and the Enzian classification for endometriosis: Their strengths and weaknesses. Acta Obs. Gynecol. Scand..

[58] Miniati M., Fiorillo C., Becatti M., Monti S., Bottai M., Marini C., Grifoni E., Formichi B., Bauleo C., Arcangeli C. (2010). Fibrin resistance to lysis in patients with pulmonary hypertension other than thromboembolic. Am. J. Respir. Crit. Care Med..

[59] Becatti M., Emmi G., Silvestri E., Bruschi G., Ciucciarelli L., Squatrito D., Vaglio A., Taddei N., Abbate R., Emmi L. (2016). Neutrophil Activation Promotes Fibrinogen Oxidation and Thrombus Formation in Behçet Disease. Circulation.

[60] Becatti M., Fiorillo C., Gori A.M., Marcucci R., Paniccia R., Giusti B., Violi F., Pignatelli P., Gensini G.F., Abbate R. (2013). Platelet and leukocyte ROS production and lipoperoxidation are associated with high platelet reactivity in Non-ST elevation myocardial infarction (NSTEMI) patients on dual antiplatelet treatment. Atherosclerosis.

[61] Becatti M., Fucci R., Mannucci A., Barygina V., Mugnaini M., Criscuoli L., Giachini C., Bertocci F., Picone R., Emmi G. (2018). A Biochemical Approach to Detect Oxidative Stress in Infertile Women Undergoing Assisted Reproductive Technology Procedures. Int. J. Mol. Sci..

[62] Whittaker A., Sofi F., Luisi M.L., Rafanelli E., Fiorillo C., Becatti M., Abbate R., Casini A., Gensini G.F., Benedettelli S. (2015). An organic khorasan wheat-based replacement diet improves risk profile of patients with acute coronary syndrome: A randomized crossover trial. Nutrients.

[63] Emmi G., Bettiol A., Niccolai E., Ramazzotti M., Amedei A., Pagliai G., Taddei N., Sofi F., Fiorillo C., Prisco D. (2021). Butyrate-Rich Diets Improve Redox Status and Fibrin Lysis in Behçet’s Syndrome. Circ. Res..

[64] Greenfield N.J. (2006). Using circular dichroism spectra to estimate protein secondary structure. Nat. Protoc..

[65] Yang Y., Li J., Chen H., Feng W. (2022). Assessment of Risk Factors Associated with Severe Endometriosis and Establishment of Preoperative Prediction Model. Diagnostics.

[66] Wiegers H.M.G., Scheres L.J.J., Tahir L., Hutten B.A., Middeldorp S., Mijatovic V. (2022). Risk of venous thromboembolism in women with endometriosis. Thromb. Res..

[67] Smyk J.M., Danielecka Z., Kotowska M., Zawadka M., Andruszkiewicz P., Grąt M., Główczyńska R., Grabowski M., Gąsecka A., Romejko-Wolniewicz E. (2024). Cardiovascular risks and endothelial dysfunction in reproductive-age women with endometriosis. Sci. Rep..

[68] Taylor H.S., Kotlyar A.M., Flores V.A. (2021). Endometriosis is a chronic systemic disease: Clinical challenges and novel innovations. Lancet.

[69] Marchandot B., Curtiaud A., Matsushita K., Trimaille A., Host A., Faller E., Garbin O., Akladios C., Jesel L., Morel O. (2022). Endometriosis and cardiovascular disease. Eur. Heart J. Open.

[70] Mu F., Rich-Edwards J., Rimm E.B., Spiegelman D., Missmer S.A. (2016). Endometriosis and Risk of Coronary Heart Disease. Circ. Cardiovasc. Qual. Outcomes.

[71] Sherwani S., Khan M.W.A., Rajendrasozhan S., Al-Motair K., Husain Q., Khan W.A. (2024). The vicious cycle of chronic endometriosis and depression-an immunological and physiological perspective. Front. Med..

[72] Adilbayeva A., Kunz J. (2024). Pathogenesis of Endometriosis and Endometriosis-Associated Cancers. Int. J. Mol. Sci..

[73] Scutiero G., Iannone P., Bernardi G., Bonaccorsi G., Spadaro S., Volta C.A., Greco P., Nappi L. (2017). Oxidative Stress and Endometriosis: A Systematic Review of the Literature. Oxid. Med. Cell. Longev..

[74] Cacciottola L., Donnez J., Dolmans M.M. (2021). Can Endometriosis-Related Oxidative Stress Pave the Way for New Treatment Targets?. Int. J. Mol. Sci..

[75] Potere N., Bonaventura A., Abbate A. (2024). Novel Therapeutics and Upcoming Clinical Trials Targeting Inflammation in Cardiovascular Diseases. Arter. Thromb. Vasc. Biol..

[76] Viganò P., Ottolina J., Sarais V., Rebonato G., Somigliana E., Candiani M. (2018). Coagulation Status in Women With Endometriosis. Reprod. Sci..

[77] Rafi U., Ahmad S., Bokhari S.S., Iqbal M.A., Zia A., Khan M.A., Roohi N. (2021). Association of Inflammatory Markers/Cytokines with Cardiovascular Risk Manifestation in Patients with Endometriosis. Mediat. Inflamm..

[78] Abramiuk M., Grywalska E., Małkowska P., Sierawska O., Hrynkiewicz R., Niedźwiedzka-Rystwej P. (2022). The Role of the Immune System in the Development of Endometriosis. Cells.

[79] Lu X., Wu Z., Wang M., Cheng W. (2018). Effects of vitamin C on the outcome of in vitro fertilization-embryo transfer in endometriosis: A randomized controlled study. J. Int. Med. Res..

[80] Nasiri N., Moini A., Eftekhari-Yazdi P., Karimian L., Salman-Yazdi R., Arabipoor A. (2017). Oxidative Stress Statues in Serum and Follicular Fluid of Women with Endometriosis. Cell J..

[81] Amreen S., Kumar P., Gupta P., Rao P. (2019). Evaluation of Oxidative Stress and Severity of Endometriosis. J. Hum. Reprod. Sci..

[82] Becatti M., Emmi G., Bettiol A., Silvestri E., Di Scala G., Taddei N., Prisco D., Fiorillo C. (2019). Behçet’s syndrome as a tool to dissect the mechanisms of thrombo-inflammation: Clinical and pathogenetic aspects. Clin. Exp. Immunol..

[83] Roitman E.V., Azizova O.A., Morozov Y.A., Aseichev A.V. (2004). Effect of oxidized fibrinogens on blood coagulation. Bull. Exp. Biol. Med..

[84] Štikarová J., Kotlín R., Riedel T., Suttnar J., Pimková K., Chrastinová L., Dyr J.E. (2013). The effect of reagents mimicking oxidative stress on fibrinogen function. Sci. World J..

[85] Rosenfeld M.A., Wasserman L.A., Vasilyeva A.D., Podoplelova N.A., Panteleev M.A., Yurina L.V. (2021). Hypochlorite-induced oxidation of fibrinogen: Effects on its thermal denaturation and fibrin structure. Biochim. Biophys. Acta Gen. Subj..

[86] Piróg M., Kacalska-Janssen O., Jach R., Ząbczyk M., Natorska J. (2021). Fibrin clot properties among women with endometriosis and the impact of ovarian stimulation. Reprod. Biomed. Online.

[87] Becatti M., Marcucci R., Bruschi G., Taddei N., Bani D., Gori A.M., Giusti B., Gensini G.F., Abbate R., Fiorillo C. (2014). Oxidative modification of fibrinogen is associated with altered function and structure in the subacute phase of myocardial infarction. Arter. Thromb. Vasc. Biol..

[88] Nowak P., Zbikowska H.M., Ponczek M., Kolodziejczyk J., Wachowicz B. (2007). Different vulnerability of fibrinogen subunits to oxidative/nitrative modifications induced by peroxynitrite: Functional consequences. Thromb. Res..

[89] Nowak W., Treliński J., Chojnowski K., Matczak J., Robak M., Misiewicz M., Nowak P. (2017). Assessment of oxidative/nitrative modifications of plasma proteins, selected ROTEM parameters and kinetics of fibrinogen polymerization in patients with multiple myeloma at diagnosis. Med. Oncol..

[90] Ceznerová E., Kaufmanová J., Stikarová J., Pastva O., Loužil J., Chrastinová L., Suttnar J., Kotlín R., Dyr J.E. (2022). Thrombosis-associated hypofibrinogenemia: Novel abnormal fibrinogen variant FGG c.8G>A with oxidative posttranslational modifications. Blood Coagul. Fibrinolysis.

[91] Torbitz V.D., Bochi G.V., de Carvalho J.A., de Almeida Vaucher R., da Silva J.E., Moresco R.N. (2015). In vitro oxidation of fibrinogen promotes functional alterations and formation of advanced oxidation protein products, an inflammation mediator. Inflammation.

[92] Isik B., Ceylan A., Isik R. (2007). Oxidative stress in smokers and non-smokers. Inhal. Toxicol..

[93] Robertson M., Chung W., Liu D., Seagar R., O’Halloran T., Koshy A.N., Horrigan M., Farouque O., Gow P., Angus P. (2021). Cardiac Risk Stratification in Liver Transplantation: Results of a Tiered Assessment Protocol Based on Traditional Cardiovascular Risk Factors. Liver Transpl..

[94] Jin K.B., Hwang E.A., Han S.Y., Park S.B., Kim H.C., Ha E.Y., Suh S.I., Mun K.C. (2008). Effects of tacrolimus on antioxidant status and oxidative stress in glioma cells. Transplant. Proc..

[95] Kwasny-Krochin B., Gluszko P., Undas A. (2010). Unfavorably altered fibrin clot properties in patients with active rheumatoid arthritis. Thromb. Res..

[96] Undas A., Kolarz M., Kopeć G., Tracz W. (2008). Altered fibrin clot properties in patients on long-term haemodialysis: Relation to cardiovascular mortality. Nephrol. Dial. Transpl..

[97] Czubkowski P., Socha P., Pawlowska J. (2011). Oxidative stress in liver transplant recipients. Ann. Transplant..

[98] Tsai Y.F., Liu F.C., Sung W.C., Lin C.C., Chung P.C., Lee W.C., Yu H.P. (2014). Ischemic reperfusion injury-induced oxidative stress and pro-inflammatory mediators in liver transplantation recipients. Transpl. Proc..

[99] Jóźwik-Plebanek K., Prejbisz A., Wypasek E., Pręgowska-Chwała B., Hanus K., Kaszuba A.M., Januszewicz M., Bieleń P., Kabat M., Kruk M. (2017). Altered plasma fibrin clot properties in hypertensive patients with obstructive sleep apnoea are improved by continuous positive airway pressure treatment. J. Hypertens..

[100] Swanepoel A.C., Lindeque B.G., Swart P.J., Abdool Z., Pretorius E. (2014). Estrogen causes ultrastructural changes of fibrin networks during the menstrual cycle: A qualitative investigation. Microsc. Res. Techol..

[101] Swanepoel A.C., Visagie A., de Lange Z., Emmerson O., Nielsen V.G., Pretorius E. (2016). The clinical relevance of altered fibrinogen packaging in the presence of 17β-estradiol and progesterone. Thromb. Res..

[102] Piróg M., Jach R., Undas A. (2017). Effects of ultra-low-dose versus standard hormone therapy on fibrinolysis and thrombin generation in postmenopausal women. Eur. J. Obs. Gynecol. Reprod. Biol..

[103] White R.E., Gerrity R., Barman S.A., Han G. (2010). Estrogen and oxidative stress: A novel mechanism that may increase the risk for cardiovascular disease in women. Steroids.

[104] Xiang D., Liu Y., Zhou S., Zhou E., Wang Y. (2021). Protective Effects of Estrogen on Cardiovascular Disease Mediated by Oxidative Stress. Oxid. Med. Cell. Longev..

[105] Iorga A., Cunningham C.M., Moazeni S., Ruffenach G., Umar S., Eghbali M. (2017). The protective role of estrogen and estrogen receptors in cardiovascular disease and the controversial use of estrogen therapy. Biol. Sex. Differ..

[106] Vannuccini S., Clemenza S., Rossi M., Petraglia F. (2022). Hormonal treatments for endometriosis: The endocrine background. Rev. Endocr. Metab. Disord..

[107] Cagnacci A., Gazzo I., Stigliani S., Paoletti A.M., Anserini P., Londero A.P., Xholli A. (2023). Oxidative Stress: The Role of Estrogen and Progesterone. J. Clin. Med..

[108] Oală I.E., Mitranovici M.I., Chiorean D.M., Irimia T., Crișan A.I., Melinte I.M., Cotruș T., Tudorache V., Moraru L., Moraru R. (2024). Endometriosis and the Role of Pro-Inflammatory and Anti-Inflammatory Cytokines in Pathophysiology: A Narrative Review of the Literature. Diagnostics.

[109] Jiang L., Yan Y., Liu Z., Wang Y. (2016). Inflammation and endometriosis. Front. Biosci. (Landmark Ed).

